# A Recombinant Chimeric Cedar Virus-Based Surrogate Neutralization Assay Platform for Pathogenic Henipaviruses

**DOI:** 10.3390/v15051077

**Published:** 2023-04-28

**Authors:** Moushimi Amaya, Randy Yin, Lianying Yan, Viktoriya Borisevich, Bishwo N. Adhikari, Andrew Bennett, Francisco Malagon, Regina Z. Cer, Kimberly A. Bishop-Lilly, Antony S. Dimitrov, Robert W. Cross, Thomas W. Geisbert, Christopher C. Broder

**Affiliations:** 1 Department of Microbiology and Immunology, Uniformed Services University, Bethesda, MD 20814, USA; 2 Henry M. Jackson Foundation for the Advancement of Military Medicine Inc., Bethesda, MD 20814, USA; 3 Department of Microbiology and Immunology, University of Texas Medical Branch, Galveston, TX 77555, USA; 4 Galveston National Laboratory, University of Texas Medical Branch, Galveston, TX 77555, USA; 5 Genomics and Bioinformatics Department, Biological Defense Research Directorate, Naval Medical Research Command–Frederick, Fort Detrick, Frederick, MD 21702, USA; 6 Defense Threat Reduction Agency, Fort Belvoir, VA 22060, USA; 7 Leidos, Inc., Reston, VA 20190, USA

**Keywords:** Hendra virus, Nipah virus, Cedar virus, henipavirus, chimera, reverse genetics, virus neutralization, vaccine, virus-host cell interaction, antibody, serum

## Abstract

The henipaviruses, Nipah virus (NiV), and Hendra virus (HeV) can cause fatal diseases in humans and animals, whereas Cedar virus is a nonpathogenic henipavirus. Here, using a recombinant Cedar virus (rCedV) reverse genetics platform, the fusion (F) and attachment (G) glycoprotein genes of rCedV were replaced with those of NiV-Bangladesh (NiV-B) or HeV, generating replication-competent chimeric viruses (rCedV-NiV-B and rCedV-HeV), both with and without green fluorescent protein (GFP) or luciferase protein genes. The rCedV chimeras induced a Type I interferon response and utilized only ephrin-B2 and ephrin-B3 as entry receptors compared to rCedV. The neutralizing potencies of well-characterized cross-reactive NiV/HeV F and G specific monoclonal antibodies against rCedV-NiV-B-GFP and rCedV-HeV-GFP highly correlated with measurements obtained using authentic NiV-B and HeV when tested in parallel by plaque reduction neutralization tests (PRNT). A rapid, high-throughput, and quantitative fluorescence reduction neutralization test (FRNT) using the GFP-encoding chimeras was established, and monoclonal antibody neutralization data derived by FRNT highly correlated with data derived by PRNT. The FRNT assay could also measure serum neutralization titers from henipavirus G glycoprotein immunized animals. These rCedV chimeras are an authentic henipavirus-based surrogate neutralization assay that is rapid, cost-effective, and can be utilized outside high containment.

## 1. Introduction

The bat-borne highly pathogenic Hendra virus (HeV) and Nipah virus (NiV) are the prototype members of the genus *Henipavirus* within the family *Paramyxoviridae* [1]. HeV and NiV are classified as Biosafety Level-4 (BSL-4) pathogens because of their high lethality and lack of approved vaccines or antivirals and are transboundary agents of significant disease threats to livestock and people in Australia and South and Southeast Asia, respectively. The genus now includes nine other reported henipaviruses; the four viral isolates of Cedar virus (CedV), Gamak virus, Daeryong virus, and Langya virus (LayV), [2,3,4] and five additional species known only from nucleic acid sequence information; Ghana bat virus (GhV), Mòjiāng virus, Melian virus, Denwin virus, and Angavokely virus (AngV) [5,6,7,8]. The recognized or apparent natural reservoir of all isolates of NiV, HeV, and CedV, along with the genomic data of GhV and AngV, are old-world fruit bats of the family *Pteropodidae*. Whereas the six other reported henipaviruses are, or are likely, of rodent origins, including the isolate LayV. Only HeV and NiV are known to be associated with severe and often fatal henipaviral disease in humans and a number of animal species (reviewed in: [9,10]), while LayV was associated with nonfatal febrile illnesses in humans [4]. In contrast, CedV is the only henipavirus isolate demonstrated to be nonpathogenic in well-established animal models of NiV and HeV infection and disease, including guinea pigs, ferrets, hamsters [2,11], and African green monkeys (Geisbert, T.W. and Broder, C.C., unpublished). An important distinction between CedV and other henipaviruses lies within the *P* gene, which encodes the phosphoprotein (P), and the *P* gene transcripts of NiV and HeV undergo RNA editing to produce the V and W nonstructural proteins that are key interferon (IFN) antagonists (reviewed in [12,13]). The CedV *P* gene lacks both RNA editing and does not encode V or W [2,14]. Several studies with recombinant NiV variants have demonstrated the differential importance of the V and W proteins in the pathogenesis brought about by NiV infection in both the hamster and ferret models, and a lack of the V protein resulted in nonlethal infections [15,16,17,18]. All other recognized or proposed henipaviruses have the potential to express V and W proteins based on current genetic data. The absence of these proteins in CedV is hypothesized to be the key factor underlying its nonpathogenic nature in established NiV and HeV animal models. These data permitted the rescue and characterization of recombinant CedV (rCedV) by reverse genetics at BSL-2 containment, and CedV is now recognized as a BSL-2 restricted agent [19,20,21].

The development of effective countermeasures against NiV and HeV has been a research priority since their discovery [22]. NiV and henipaviral diseases are also included on the WHO’s Blueprint List of Priority Pathogens [23], and NiV is among the Coalition for Epidemic Preparedness Innovations (CEPI) list of Priority Diseases needing urgent research and countermeasure development [24,25]. There are currently no licensed NiV/HeV vaccines or antivirals approved for human use, although a licensed vaccine to prevent HeV infection in horses, based on a soluble form of the attachment (G) glycoprotein (sG) from HeV (HeV-sG), was launched in Australia (Equivac^®^ HeV) by Zoetis, Inc., in November 2012 [26]. A NiV vaccine formulated for human use with the HeV-sG immunogen is currently in Phase 1 human clinical trials [27]. As a therapeutic approach development for henipavirus human infection, the human monoclonal antibody (mAb) m102.4, specific to the NiV and HeV G glycoprotein receptor binding site, has completed a Phase I clinical trial in Australia [28]. To date, 18 individuals exposed to either HeV in Australia (*n* = 17) or NiV in the United States (*n* = 1) have received high-dose, post-exposure, m102.4 therapy (15–20 mg/kg) by emergency use protocols and no evidence of virus infection has been reported.

The G glycoprotein (also referred to as the receptor-binding protein (RBP)), together with the fusion (F) glycoprotein on the surface of the henipavirus virion, are the mediators of virus attachment and infection [29]. The RBP determines the cellular tropism of infection. The NiV and HeV G glycoproteins specifically bind to cells expressing the ephrin-B class ligands, ephrin-B2, and ephrin-B3 [30,31,32,33]. In contrast, CedV has a uniquely broad ephrin protein tropism and can utilize both B-class and A-class ephrins for cell entry and infection [21,34]. The binding of NiV or HeV G to their ephrin entry receptors on cells triggers a well-characterized activation and refolding of F from a pre- to post-fusion conformation that facilitates the merger of the virion and host cell membranes and subsequent delivery of the viral nucleocapsid into the cell cytoplasm (reviewed in [35]). Importantly, the F and G glycoproteins are also the major viral structural protein targets of neutralizing antibodies and the relevant antigens employed in all henipavirus vaccine strategies [22]. However, for pathogenic henipavirus vaccines or antibody-based countermeasure strategies, the assessment and quantification of neutralizing antibody responses or their potencies against authentic NiV and HeV, which requires BSL-4 containment, can be a major challenge.

Using a rCedV reverse genetics platform, the CedV F and G glycoprotein genes were replaced with those of NiV-B or HeV, and replication-competent chimeric henipaviruses (rCedV-NiV-B and rCedV-HeV) were rescued. Both non-reporter and two reporter gene versions, encoding a green fluorescent protein (GFP) or luciferase protein (Luc), of the rCedV chimeras were also produced. Characterization of the chimeric viruses revealed no significant differences in their replication kinetics or ability to induce a type I IFN response compared to rCedV and possessed the same ephrin B-class entry receptor tropisms as NiV-B and HeV. The neutralization potencies of several well-characterized cross-reactive NiV and HeV F and G specific mAbs against rCedV-NiV-B-GFP and rCedV-HeV-GFP were highly correlated with those measured using authentic NiV-B and HeV when tested in parallel by a plaque reduction neutralization test (PRNT). A rapid high-throughput and quantitative fluorescence reduction neutralization test (FRNT) using the GFP-encoding chimeras was established that also yielded highly correlated mAb neutralization potencies with those derived by PRNT. The FRNT assay was also suitable for measuring serum neutralization titers from animals immunized with recombinant HeV or NiV soluble G glycoproteins. Taken together, the rCedV chimera platform is an authentic henipavirus-based surrogate neutralization assay for pathogenic henipaviruses that is rapid, cost-effective and can be utilized outside BSL-4 containment.

## 2. Materials and Methods

### 2.1. Construction of pOLTV5-rCedV Chimeric Antigenomes

**Figure 1:**
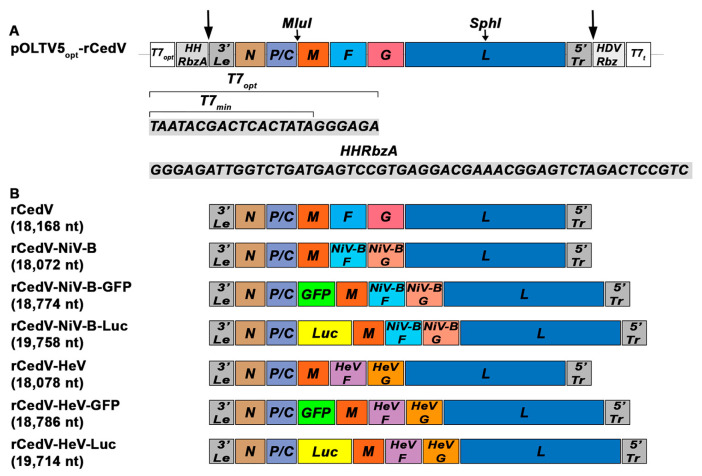
Schematic representation of the optimized rCedV plasmid and the genomes of the generated rCedV chimeric viruses. (**A**) The pOLTV5_opt_-rCedV plasmid illustrates the location and sequences of the T7 optimal promoter (T7_opt_) and the Hammerhead Ribozyme A (HHRbzA). The long arrows indicate regions of self-cleavage. Unique restriction sites MluI and SphI used to construct the rCedV chimeric plasmids are shown. (**B**) The genomes and the lengths of the generated chimeras are schematically diagrammed as rCedV-NiV-B, rCedV-NiV-B-GFP, rCedV-NiV-B-Luc, rCedV-HeV, rCedV-HeV-GFP, and rCedV-HeV-Luc. pOLTV5_opt_-rCedV, optimized pOLTV5-rCedV plasmid; T7_min_, T7 minimal promoter; T7_opt_, T7 optimal promoter; HHRbzA, Hammerhead Ribozyme A; 3′Le, 3′ Leader; 5′Tr, 5′ Trailer; HDVRbz, hepatitis delta virus ribozyme; T7_t_, T7 terminator.

A full description of the synthesis of the rCedV antigenome clone (pOLTV5-rCedV) has previously been described [20]. Here, an optimized version of the pOLTV5-rCedV antigenome clone was designed, and the 3′ end of the T7 minimal promoter (T7_min_) sequence (TAATACGACTCACTATA) was modified by the addition of nucleotides GGGAGA to generate a T7 optimal promoter (T7_opt_) [36]. The T7_opt_ sequence was then followed by the insertion of a self-cleaving autocatalytic hammerhead ribozyme A (HHRbzA) sequence (GGGAGATTGGTCTGATGAGTCCGTGAGGACGAAACGGAGTCTAGACTCCGTC) [36]. This synthesized gene fragment (T7_opt_-HHRbzA) (Genscript; NJ, USA) was enzymatically inserted to precede the CedV 3′ Leader (3′ Le) sequence in the pOLTV5-rCedV plasmid to yield pOLTV5_opt_-rCedV (Figure 1A).

Large gene cassettes comprising CedV F and G untranslated intergenic regions flanking the respective NiV-B or HeV F and G coding sequences were synthesized (Genscript). The NiV-B F and G coding sequences were based on the NiV-B 2010 Faridpur isolate (GenBank: JN808864.1). The HeV F and G protein sequences used here, HeV genome (GenBank: MN062017.1), are identical to that of the HeV 2008 Redlands isolate (GenBank: JN255805.1). The isolates and GenBank accession numbers for each F and G protein are listed in Table 1. Unique restriction enzyme sites facilitated the insertion of the NiV-B or HeV F and G gene cassettes into pOLTV5_opt_-rCedV (Figure 1A) to ultimately generate non-reporter versions, pOLTV5_opt_-rCedV-NiV-B or pOLTV5_opt_-rCedV-HeV. The “rule-of-six” was maintained by removing the last three nucleotides (ACG; amino acid Threonine) from the NiV-B F coding sequence and adding a stop codon (TAA) to the end of the HeV F coding region. The insertion of a modified turbo Green Fluorescent Protein (*GFP*) gene (Lonza Inc., Allendale, NJ, USA) or firefly (*Photinus pyralis*) luciferase (*Luc*) gene (a kind gift from Dr. B Schaefer, USU) between CedV *P* and *M* genes of the newly constructed pOLTV5_opt_-rCedV chimeric plasmids using standard molecular techniques was as previously described [20,37] and yielded pOLTV5_opt_-rCedV chimeric reporter gene encoding versions of the rCedV antigenome clones. All cloning was performed with *Escherichia coli* Stbl2 cells (Invitrogen; Carlsbad, CA, USA). The insertions were sequenced to obtain at least 2-fold coverage.

### 2.2. Cells, Monoclonal Antibodies, Rhesus Macaque, and Rabbit Immune Sera

BSR-T7/5 cells, a BHK-derived cell line stably expressing T7 RNA polymerase [38], HeLa-USU, HeLa (ATCC CCL-2), Vero E6 (ATCC CCL-81), and Vero 76 (ATCC CRL-1587) cell lines were maintained at 37 °C, 5% CO_2_ in Dulbecco’s modified eagle media (DMEM) (Quality Biological; Gaithersburg, MD, USA) supplemented with 10% cosmic calf serum (CCS) and 1% L-glutamine (Quality Biological) (DMEM-10). HeLa-USU-ephrin-B2 and HeLa-USU-ephrin-B3 stable cell lines were maintained in DMEM-10% CCS, 1% L-glutamine supplemented with 0.4 mg/mL Hygromycin B (Invitrogen).

The neutralizing HeV and NiV cross-reactive human mAb, m102.4, is a G glycoprotein-specific IgG1 subclass antibody [28,39,40,41,42,43]. The humanized 5B3.1 (h5B3.1) mAb [44,45] and the murine mAbs 12B2 and 1F5 [46] are IgG1 mAbs cross-reactive to HeV and NiV F glycoprotein. Anti-NiV G glycoprotein-specific sera were from four rhesus macaques immunized at the University of Texas Medical Branch at Galveston, TX, on days 0, 28, and 56 with an equal mixture of 0.1 mg NiV-B and 0.1 mg NiV-M recombinant soluble G (sG) glycoproteins adjuvanted with aluminum hydroxide suspension (Auro Vaccines, LLC, Pearl River, NY, USA) [47]. Sera were collected on days 42 and 84 post-immunization and stored at −80 °C. Anti-HeV G glycoprotein-specific sera were prepared using the HeV/NiV recombinant soluble HeV G glycoprotein vaccine candidate (HeV-sG) [26,27,48,49,50,51,52,53,54] or HeV-sG_tet_ [55], a tetrameric version of the HeV sG glycoprotein which was constructed similarly to other henipavirus sG glycoproteins as described in Cheliout Da Silva et al. [56]. Sera from rabbits immunized on days 0 and 28 with 0.1 mg HeV-sG or HeV-sG_tet_ formulated with complete Freund’s adjuvant (initial injection) and boosted with immunogen formulated with incomplete Freund’s adjuvant (booster injection) were prepared by Noble Life Sciences; Woodbine, MD. Sera were collected on day 45 post-immunization and stored at −80 °C.

### 2.3. Rescue of Recombinant CedV Chimeras

BSR-T7/5 cells in a 12-well plate (2.5 × 10^5^ cells/well) were co-transfected with pCMV-CedV helper plasmids pCMV-CedV-N (1.25 µg), pCMV-CedV-P (0.8 µg) and pCMV-CedV-L (0.4 µg) together with one of the pOLTV5_opt_-rCedV chimera antigenome constructs (3.5 µg) using TransIT-LT1 transfection reagent (Mirus Bio; Madison, WI, USA) according to the manufacturer’s recommendations. After 4–5 days, transfected cells were observed for syncytia formation and/or GFP expression. Supernatants from successful rescue wells were collected and passaged onto naïve Vero E6 cells in a T-75 flask to prepare a master stock of each of the rCedV chimeras. When maximal syncytia and/or GFP expression was observed (~2–3 days), viral supernatants were collected and clarified by centrifugation at 948× *g* (2400 rpm) for 10 min to pellet cell debris. The supernatant was transferred to screw-cap tubes as single-use aliquots and stored at −80 °C. All rCedV chimeras were deep sequenced using Illumina short reads, and variants were analyzed. Briefly, sequencing libraries were prepared from total RNA using the TruSeq Stranded Total RNA Sample Prep kit (Illumina, San Diego, CA, USA) and subjected to multiplexed sequencing on either the Illumina MiSeq platform using 600 cycles, V3 chemistry, or the Illumina NextSeq platform using 300 cycles, V2 chemistry. The resulting sequencing reads were analyzed using EDGE Bioinformatics tools [57] and an in-house metagenomics pipeline called MetaDetector (unpublished). EDGE Bioinformatics suite was used for read processing, and the host read subtraction, de novo assembly, taxonomic classification, and variant detection. Sequencing reads were also processed in parallel using MetaDetector, which checked for quality using FASTQC [58], trimmed for quality using BBDuk (Q20) [59], and removed incidental matching human genome reads using BBMAP [59]. The remaining reads were assembled using metaSPAdes and SPAdes [60]; the resulting contigs, along with all the cleaned singleton reads, were BLAST searched using Diamond [61] for taxonomic classification. The final assemblies were examined and constructs were identified to be annotated using BLASTn and BLASTx implemented in CLC Genomics Workbench (QIAGEN Bioinformatics; Redwood City, CA). Variant analysis was performed by mapping the reads to a publicly available reference Cedar virus genome from isolate CG1a (GenBank accession JQ001776) using EDGE Bioinformatics tools and iVar [62].

### 2.4. Viral Plaque Assay

Viral stocks were titrated by plaque assay as previously described [20,37,63]. Briefly, a ten-fold serial dilution of the virus stock was prepared in DMEM-10, 200 µL of which was applied to pre-seeded Vero E6 cells in duplicate (5 × 10^5^ cells/well) in a 12-well plate and incubated for 1 h at 37 °C, 5% CO_2_. A 2 mL overlay of a 1:1 mix of DMEM containing 5% CCS and 1% L-glutamine (DMEM-5) with 2% carboxymethylcellulose sodium salt (medium viscosity) (Sigma-Aldrich; St. Louis, MO, USA) was applied to all wells and incubated for 4 days at 37 °C, 5% CO_2_. Cells were fixed with 4% Formaldehyde in 1× PBS for 1 h at room temperature and stained with 0.5% crystal violet in 80% methanol for 15 min at room temperature. The stain was removed and washed with diH_2_O, and plaque-forming units (PFU) were counted and expressed as PFU/mL.

### 2.5. Virus Biosafety Procedures and Regulations

Laboratory manipulation guidelines and standard operating procedures for rCedV-NiV-B, rCedV-NiV-B-GFP, rCedV-NiV-B-Luc, rCedV-HeV, rCedV-HeV-GFP, rCedV-HeV-Luc, rCedV, rCedV-GFP and rCedV-Luc under BSL-2 conditions have been established, reviewed, and approved by the Uniformed Services University (USU), Institutional Biosafety Committee in accordance with NIH guidelines. The rCedV-HeV-GFP and/or rCedV-NiV-B-GFP chimeras have been previously used in mAb neutralization and mAb synergy neutralization studies [47,64,65]. A P2 stock of NiV-B was used in these studies. There were four mutations of sufficient frequency in comparison to the reference sequence GenBank Accession number AY988601.1. Of these, one was non-coding, and the other three led to single amino acid changes: one in the M protein and two in the F protein [66]. The HeV isolate used in these studies (GenBank Accession number NC_001906) was obtained from a patient from the 1994 outbreak in Australia and was provided by Dr. Thomas Ksiazek [45]. All studies with authentic NiV-B and HeV were performed within the BSL-4 facilities of the Galveston National Laboratory, The University of Texas Medical Branch at Galveston, TX, USA.

### 2.6. Western Blot Analysis

Vero E6 cells in a 6-well plate were infected at a density of 1 × 10^6^ cells/well with rCedV-NiV-B, rCedV-NiV-B-GFP, rCedV-NiV-B-Luc, rCedV-HeV, rCedV-HeV-GFP, rCedV-HeV-Luc, rCedV, rCedV-GFP or rCedV-Luc at a multiplicity of infection (MOI) of 0.01. Simultaneously, the cells were co-transfected with a total of 2 µg of the plasmid to express both F and G glycoproteins from either NiV or HeV. A promoter-modified pcDNA3.1 vector with a hygromycin selection marker [67] encoding either NiV-F (pcDNA3.1-NiV-F) or NiV-G (pcDNA3.1-NiV-G) was used for the NiV-F and NiV-G expression. A promoter-modified pcDNA3.1 encoding either HeV-F (pcDNA3.1-HeV-F) or HeV-G (pcDNA3.1-HeV-G) was used for the HeV-F and HeV-G expression. At 24 h and 48 h post-infection, cells were collected and lysed with 1× RIPA (radioimmunoprecipitation assay) Lysis and Extraction Buffer (ThermoFisher Scientific; Waltham, MA, USA) containing a protein inhibitor cocktail (ThermoFisher Scientific). Total protein (~30 µg) in reducing sample buffer (2× lithium dodecyl sulfate (LDS) NuPage^®^ sample buffer (Invitrogen), 5% β-mercaptoethanol (Sigma-Aldrich, St. Louis, MO, USA) was boiled for 10 min at 100 °C. Proteins were separated by sodium dodecyl sulfate-polyacrylamide gel electrophoresis (SDS-PAGE) on a 4–12% Bis-Tris gel (ThermoFisher Scientific) and then transferred on nitrocellulose membranes (ThermoFisher Scientific). The membranes were blocked in 5% milk in 1× PBS with 0.1% Tween-20 at room temperature. Cross-reactive murine mAbs specific to NiV and HeV F (mAb 5G7) or to NiV and HeV G (mAb 48D3) glycoproteins, polyclonal rabbit sera to CedV-N (CSIRO, Victoria, Australia), and β-actin (ThermoFisher Scientific) were used as primary antibodies and subsequently probed with a corresponding secondary HRP-coupled antibody.

### 2.7. Virus Replication Kinetics

Vero E6 cells were seeded at a density of 2 × 10^4^ cells/well in a 96-well cell culture plate. The next day, cells were infected at an MOI of 0.01 at 37 °C, 5% CO_2_. After 1 h, the viral inoculum was removed, and fresh DMEM-10 was added to all wells. Supernatants were collected at 0, 8, 24, 48, and 72 h post-infection and stored at −80 °C until ready to analyze. Viral titers were determined by plaque assay as described in Section 2.4 and were expressed as plaque-forming units (PFU) per mL (PFU/mL). To determine intracellular luciferase activity, cells infected with either the non-reporter or Luc expressing viral chimeras were lysed with the Steady-Glo^®^ Luciferase Assay System (Promega, Madison, WI, USA) in a 1:1 mixture with DMEM-10. After a 10 min incubation at room temperature, the homogenate was transferred to a white opaque 96-well cell culture plate, Nunc™ F96 MicroWell™ White Polystyrene Plate (ThermoFisher Scientific), and luminescence read using the GloMax^®^—Multi Detection System (Promega, Madison, WI, USA). Relative light units (RLU) were measured and normalized by subtracting the luminescence values of rCedV-NiV-B or rCedV-HeV infected cells from the luminescence values of rCedV-NiV-B-Luc or rCedV-HeV-Luc infected cells, respectively. Virus titers and luciferase activity levels at 0 h post-infection indicate the lower limit of detection for the plaque assay and the luminometer, respectively.

### 2.8. Ephrin Entry Receptor Tropism

HeLa-USU, HeLa-USU-ephrin-B2, and HeLa-USU-ephrin-B3 cell lines were seeded at a density of 2.5 × 10^5^ cells/well in a 12-well cell culture plate. When confluent, the cell culture medium was removed, and cells were left uninfected (Mock) or infected at an MOI of 0.5 with either rCedV-NiV-B-GFP, rCedV-HeV-GFP, or rCedV-GFP individually diluted in DMEM-10. At 24 h post-infection, rCedV-NiV-B-GFP, rCedV-HeV-GFP, and rCedV-GFP infected cell cultures were monitored for GFP fluorescence and syncytia. Images were captured with a Zeiss Axio Observer A1 inverted microscope using the 5× objective.

### 2.9. Reverse Transcriptase Quantitative PCR (RT-qPCR) and Type I IFN Response

HeLa-CCL-2 cells were seeded at a density of 1.25 × 10^5^ cells/well in a 24-well plate and incubated overnight. Cells were left intact (Mock), transfected with polyinosinic:polycytidylic acid (Poly I:C) (InvivoGen; San Diego, CA, USA) (10 μg/mL) using Lipofectamine LTX (ThermoFisher Scientific), or infected with rCedV-NiV-B, rCedV-HeV or rCedV at either an MOI of 0.5 or 1.0. At 24 h post-infection, total RNA was extracted using the RNeasy Mini Kit (Qiagen Sciences Inc., Germantown; MD, USA). An amount of 500 ng of DNase I digested RNA was converted to cDNA using the Superscript III First-Strand Synthesis System (ThermoFisher Scientific) with oligo(dT) primers. Quantitative PCR (qPCR) was then performed with the synthesized cDNA using the Power SYBR Green PCR Master Mix (ThermoFisher Scientific) and the Applied Biosystems 7500 Real-Time PCR System. PCR cycling conditions were: 95 °C, 10 min; 40× cycles of 95 °C, 15 s; 60 °C, 1 min; followed by a melt curve analysis at the completion of each experiment. Each sample was analyzed for IFN-α, IFN-β and 18S ribosomal RNA in triplicate, and fold changes were calculated relative to 18S ribosomal RNA and normalized to mock samples using the 2^(−ΔΔCt)^ method. IFN-α forward primer, 5′ TTTCTCCTGCCTGAAGGACAG 3′, IFN-α reverse primer, 5′ ACAGTCTCGTCTTTAGTACTCG 3′ [68]. IFN-β forward primer, 5′ GTCAGAGTGGAAATCCTAAG 3′, IFN-β reverse primer, 5′ ACAGCATCTGCTGGTTGAAG 3′ [69]. An 18S ribosomal RNA forward primer 5′ GGGCATTCGTATTTCATAGTCAGAG 3′, 18S ribosomal RNA reverse primer 5′ CGGTTCTTGATTAATGAAAACATCCT 3′ [70].

### 2.10. Plaque Reduction Neutralization Test (PRNT)

Vero 76 cells were seeded at a density of 6 × 10^5^ cells/well in a 6-well plate and incubated overnight at 37 °C, 5% CO_2_. The mAbs were serially diluted 3-fold in DMEM-10 such that an initial concentration of 10 µg/mL was used for the 9-point dose-response curve. The diluted mAbs were incubated with an equal volume of either rCedV-NiV-B-GFP, rCedV-HeV-GFP, NiV-B, or HeV at an MOI of 0.0001 for 1 h at 37 °C, 5% CO_2_. MOI was calculated for a tentative 10^6^ cells/well and 0.4 mL virus and antibody mixture per well. Each virus-mAb mixture (400 µL/well) was added to duplicate wells. Following a 1 h incubation at 37 °C, 5% CO_2_, the wells were overlaid with a 1:1 mix of 0.8% agarose/DMEM-10 and incubated for 4 days. A neutral red solution was added to each well and incubated for 24 h, at which time plaques were counted. Neutralization percent (%) was calculated by subtracting the PFU_mAb_ for each virus from the respective PFU without the antibody, i.e., $Neutralization\left(\%\right)=100\times \frac{\left(PFU_{0}-PFU_{mAb}\right)}{PFU_{0}}$, where the PFU_mAb_ is the PFU at the respective mAb concentration, and PFU_0_ is the PFU without the antibody. The 50% inhibitory concentration (IC_50_) was determined as the antibody concentration at which there was a 50% reduction in plaque counts versus untreated control wells. The IC_50_ values were calculated by non-linear regression curve fitting with a variable slope using GraphPad Prism 9 (GraphPad Software Inc., San Diego, CA, USA). The limit of detection for this assay was 50 PFU.

### 2.11. Fluorescent Reduction Neutralization Test (FRNT)

Vero 76 cells were seeded at a density of 2 × 10^4^ cells/well in black-walled clear bottom 96-well plates (Corning Life Sciences; Corning, NY, USA) and incubated for 24 h at 37 °C, 5% CO_2_. m102.4, h5B3.1, 12B2, and 1F5 mAbs were serially diluted 3-fold such that an initial concentration of 1.1 µg/mL was used for the 7-point dose-response curve. Immunized sera were 3-fold serially diluted in DMEM-10 such that NiV-B and NiV-M sG immunized rhesus macaque sera were at a starting dilution of 1:200, and the HeV-sG and HeV-sG_tet_ rabbit sera were at a starting dilution of 1:400. An equal volume of DMEM-10 containing either rCedV-NiV-B-GFP or rCedV-HeV-GFP was added to each dilution for a final MOI of 0.05 and incubated for 2 h at 37 °C, 5% CO_2_. Each of the virus-mAb or virus-sera mixtures (90 µL/well) was added to the pre-seeded Vero 76 cells in triplicate and incubated for an additional 24 h at 37 °C, 5% CO_2_. The virus-mAb supernatants were removed, and the plates were fixed with 4% Formaldehyde in 1× PBS for 20 min at room temperature. The plates were then washed 3 times by hand with a slow stream of diH_2_O, and the last wash was discarded before the plates were imaged using a CTL S6 analyzer (Cellular Technology Limited; Shaker Heights, OH, USA). Fluorescent foci were counted using the CTL Basic Count software. The 50% inhibitory concentration (IC_50_) was determined as the antibody concentration or serum dilution at which there was a 50% reduction in fluorescent foci versus untreated control wells. The IC_50_ values were calculated by non-linear regression curve fitting with a variable slope using GraphPad Prism 9 (GraphPad Software Inc., San Diego, CA, USA). The limit of detection for this assay was 50 fluorescent foci.

### 2.12. Statistical Analysis

Data were analyzed and graphed using GraphPad Prism 9 (GraphPad Software Inc.). Unless otherwise stated, graphs and images are the average of three independent experiments and are expressed as the arithmetic mean. Standard deviations were calculated and represented accordingly. Statistical analyses for viral replication kinetics were performed with two-way ANOVA followed by the Tukey post hoc test (α = 0.05). Statistical analyses for qPCR experiments were performed with the unpaired, two-tailed Student *t*-test using GraphPad Prism 9. Correlation analyses were performed using Pearson correlation coefficient analyses.

## 3. Results

### 3.1. Construction and Rescue of Recombinant Cedar Virus-Based Chimeras

To generate rCedV chimeric viruses encoding the NiV-B or HeV envelope glycoprotein genes, we first optimized the virus rescue efficiency of the previously described rCedV reverse genetics system [20]. A pOLTV5_opt_-rCedV antigenome plasmid was constructed by inserting a DNA fragment containing sequences for a T7 optimal promoter (T7_opt_) and an autocatalytic Hammerhead Ribozyme A (HHRbzA) sequence upstream of the rCedV 3′ leader antigenome sequence (see Materials and Methods) (Figure 1A). Next, a large fragment of the pOLTV5_opt_-rCedV plasmid flanked by unique restriction enzyme sites MluI and SphI was replaced with synthesized DNA fragments containing the open reading frames of either the NiV-B or HeV F and G glycoproteins in place of the CedV F and G glycoprotein encoding region. Reporter genes containing antigenome plasmids encoding either *GFP* or *Luc* genes for each chimera were also generated (see Materials and Methods). The reverse genetics method was then used to rescue a panel of replication-competent rCedV chimeras as non-reporter gene versions (rCedV-NiV-B and rCedV-HeV) and reporter gene encoding versions expressing GFP (rCedV-NiV-B-GFP and rCedV-HeV-GFP) or Luc (rCedV-NiV-B-Luc and rCedV-HeV-Luc) proteins. A schematic representation of the genomes and genome lengths of all rescued viruses is illustrated in Figure 1B. Successful rescue of the viruses was confirmed by the detection of syncytia formation (cytopathic effect (CPE)) when supernatants from BSR-T7/5 cells transfected with the rCedV chimeric antigenome and CedV helper plasmids were then passaged onto Vero E6 cells. Stock virus preparations were subsequently prepared, and virus genomes were sequenced. When compared to the predicted genome sequences, the following mutations were detected within coding sequences in the chimera genomes (e.g., excluding intergenic regions) (Table S1). There were two mutations in the rCedV-HeV genome: one synonymous single nucleotide variation (SNV) and one SNV that resulted in a single amino acid change in the F protein. Three mutations were detected in the rCedV-HeV-GFP genome: one synonymous SNV and two that resulted in single amino acid changes: one in the N protein and the other in the M protein. There were four mutations in the rCedV-HeV-Luc genome, and all four were synonymous mutations. The rCedV-NiV-B genome contained four mutations, all resulting in single amino acid changes: one in the N protein, one in the M protein, and two in the L protein. There were eight mutations in the rCedV-NiV-B-GFP genome: six were synonymous SNVs, and two resulted in single amino acid changes in the N protein. There were nine mutations detected in the rCedV-NiV-B-Luc genome: five were synonymous SNVs, and four resulted in single amino acid changes: one in the N protein, one in the Luc protein, one in the M protein, and one in the L protein. No apparent loss of rCedV chimera reproductive capacity or reporter gene loss or integrity has been observed to date, probably owing to the requirement of these paramyxoviruses to the ‘rule-of-six.’

### 3.2. Characterization of Recombinant Cedar Virus-Based Chimeric Viruses

**Figure 2:**
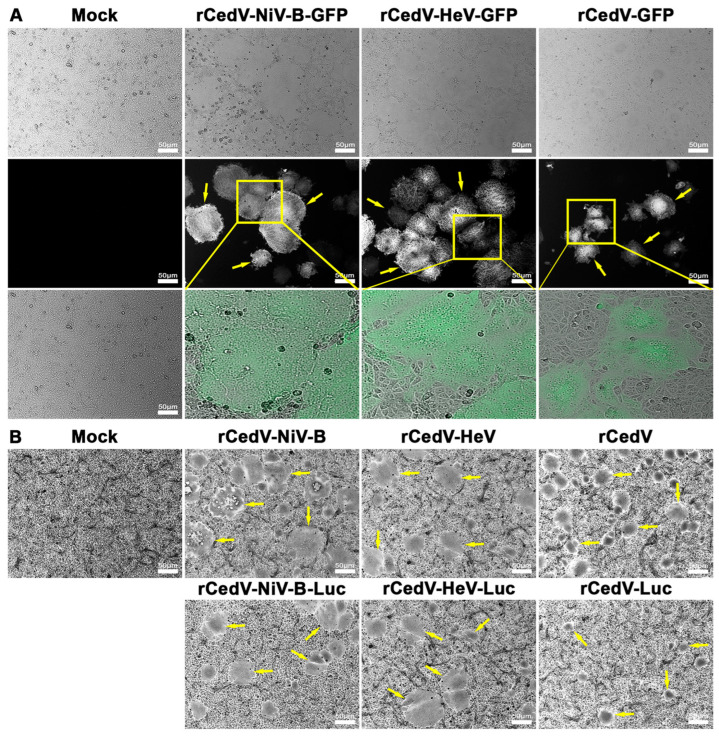
Syncytia induced by rCedV expressing NiV-B or HeV envelope glycoproteins. Vero E6 cells were uninfected (Mock) or infected with either rCedV-NiV-B, rCedV-NiV-B-GFP, rCedV-NiV-B-Luc, rCedV-HeV, rCedV-HeV-GFP, rCedV-HeV-Luc, rCedV, rCedV-GFP or rCedV-Luc at a MOI of 0.01. All images were taken 24 h post-infection. (**A**) Cells infected with GFP-expressing viruses. Transmitted light (**top** row), fluorescence (**middle** row), and merged (**bottom** row) images are shown. The respective zoomed-in fluorescence images (3rd row) are regions from the yellow boxes. (**B**) Cells infected with non-reporter or Luc expressing rCedV chimeras were fixed, stained, and then imaged for syncytia. The images taken with transmitted light are shown. Images were captured with a Zeiss Axio Observer A1 inverted microscope using a 5× objective. Arrows indicate giant multinucleated cells (syncytia). Representative images from three independent experiments are shown. Scale bar, 50 µm.

We assessed the ability of the rCedV-NiV-B chimeras and the rCedV-HeV chimeras to facilitate membrane fusion and syncytia formation when used to infect cells (Figure 2). Vero E6 cells were either uninfected (Mock) or infected with either rCedV-NiV-B, rCedV-NiV-B-GFP, rCedV-NiV-B-Luc, rCedV-HeV, rCedV-HeV-GFP, or rCedV-HeV-Luc and comparisons then made to Vero E6 cells infected with rCedV, rCedV-GFP or rCedV-Luc. At 24 h post-infection, cells infected with the GFP-expressing viruses were imaged for fluorescence and syncytia (Figure 2A), while cells infected with the non-reporter or Luc expressing rCedV chimeras were imaged following fixation and crystal violet staining (Figure 2B). Fluorescence and/or syncytia (yellow arrows) were observed in all infected Vero E6 cells. The syncytia observed in cells infected with either the rCedV-NiV-B chimeras or the rCedV-HeV chimeras were noticeably larger and contained more nuclei than those syncytia observed in rCedV-infected cells (Figure 2). These data confirmed the functionality of the NiV-B and HeV F and G glycoproteins expressed in the context of rCedV.

**Figure 3:**
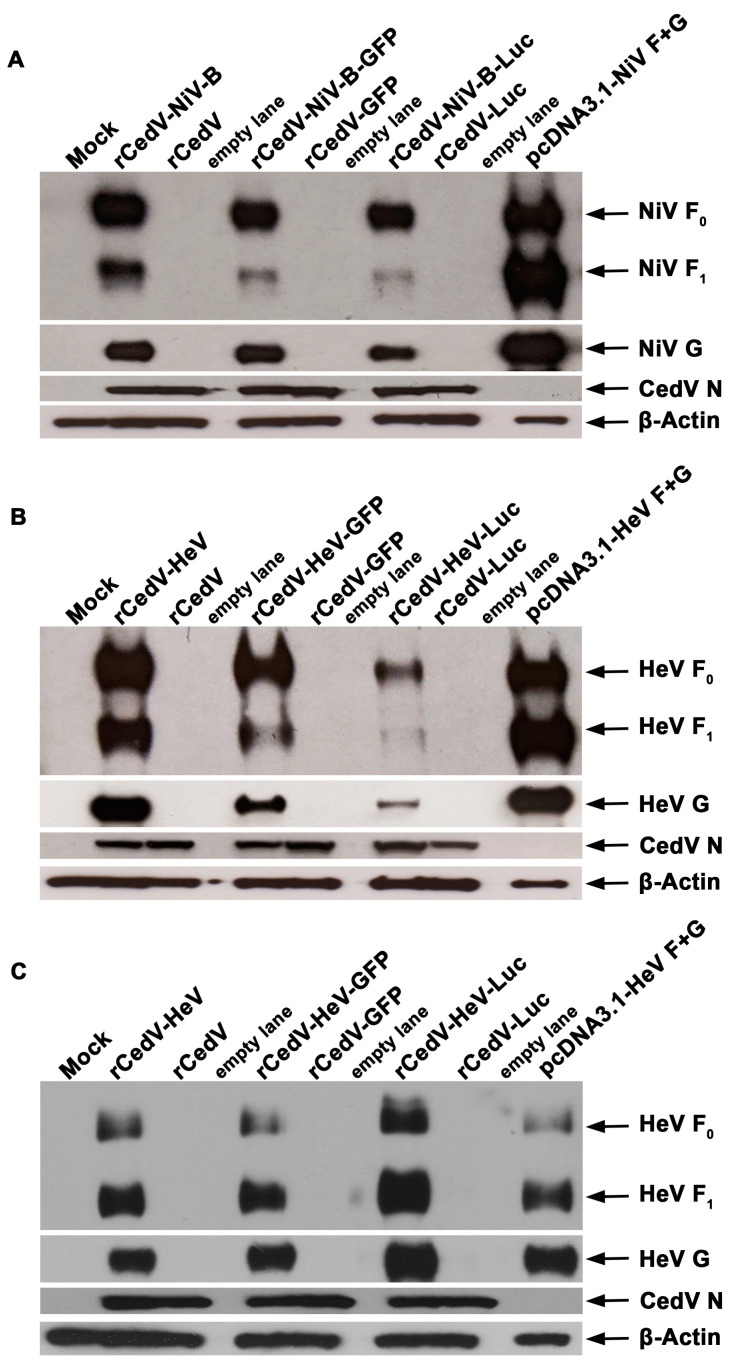
Expression of NiV-B and HeV envelope glycoproteins in infected cells. Vero E6 cells were uninfected (Mock) or infected at a MOI of 0.01 with either rCedV-NiV-B, rCedV-NiV-B-GFP, rCedV-NiV-B-Luc (**A**), rCedV-HeV, rCedV-HeV-GFP, rCedV-HeV-Luc (**B**,**C**), rCedV, rCedV-GFP or rCedV-Luc. As a reference, cells were co-transfected with a total of 2 µg of pcDNA3.1-NiV-F and pcDNA3.1-NiV-G (pcDNA3.1-NiV F + G), or pcDNA3.1-HeV-F and pcDNA3.1-HeV-G (pcDNA3.1-HeV F + G). Cells were harvested at 24 h post-infection (**A**,**B**) (rCedV-NiV-B and rCedV-HeV chimeras) or 48 h post-infection (**C**) (rCedV-HeV chimeras only), lysates were prepared and total protein (~30 µg) resolved by SDS-PAGE followed by western blot assay. The subsequent membrane was probed with HeV and NiV cross-reactive monoclonal antibodies (mAbs) against F glycoprotein (mAb 5G7) and G glycoprotein (mAb 48D3), polyclonal rabbit serum to CedV-N and β-actin. Representative images from two independent experiments are shown.

**Figure 4:**
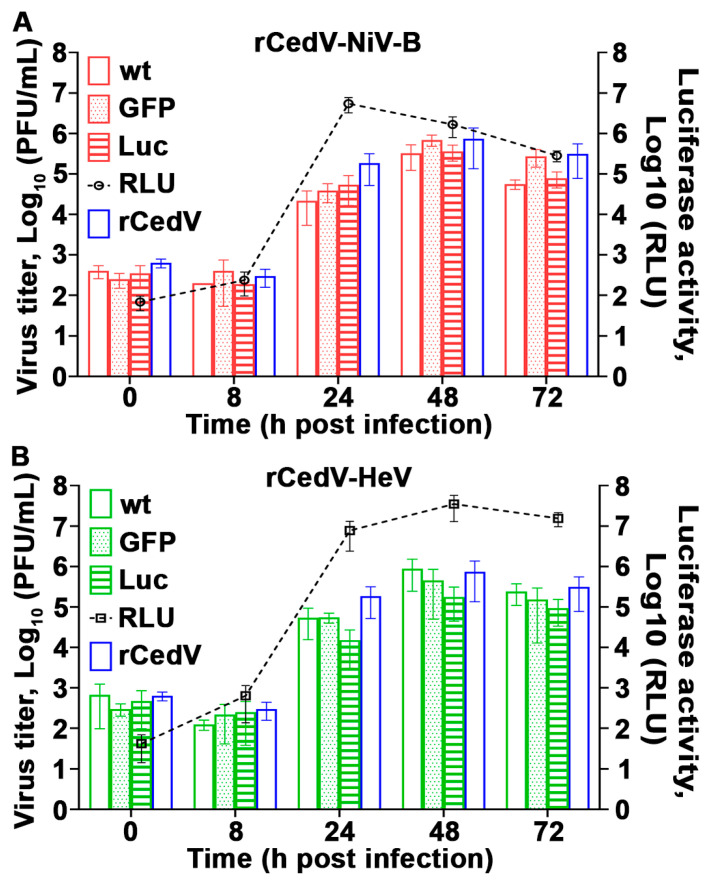
Replication kinetics of rCedV chimeras. Infectious virus titers (PFU/mL) determined from supernatants harvested at the indicated time points from Vero E6 cells infected at a MOI of 0.01 with rCedV-NiV-B (clear red bar), rCedV-NiV-B-GFP (dotted red bar), rCedV-NiV-B-Luc (striped red bar) (**A**), rCedV-HeV (clear green bar), rCedV-HeV-GFP (dotted green bar) or rCedV-HeV-Luc (striped green bar) (**B**). As a reference, separate populations of Vero E6 cells were also infected with rCedV (blue bar) (**A**,**B**). Normalized relative light units (RLU) for CedV-NiV-B-Luc (**A**) and rCedV-HeV-Luc (**B**) infected cells are represented on the right *y*-axes as black dashed lines. These data represent mean ± standard deviation from three independent experiments. Virus titers and luciferase activity levels at 0 h post-infection indicate the lower limit of detection for the plaque assay and the luminometer, respectively. Statistical analysis was performed in GraphPad Prism 9 by two-way ANOVA followed by Tukey’s post hoc test (α = 0.05).

We next evaluated the relative expression levels of NiV-B and HeV F and G envelope glycoproteins from cells infected with the rCedV chimeras in comparison to rCedV (comparative control). Vero E6 cells were uninfected (Mock) or infected with either rCedV-NiV-B, rCedV-NiV-B-GFP, rCedV-NiV-B-Luc, rCedV-HeV, rCedV-HeV-GFP, rCedV-HeV-Luc, rCedV, rCedV-GFP or rCedV-Luc. For additional comparative purposes, separate populations of Vero E6 cells were co-transfected with plasmids, pcDNA3.1-NiV-F and pcDNA3.1-NiV-G (pcDNA3.1-NiV F + G), or pcDNA3.1-HeV-F and pcDNA3.1-HeV-G (pcDNA3.1-HeV F + G). Representative western blot images for NiV-B and HeV F and G glycoproteins probed with cross-reactive NiV/HeV F or G specific mAbs are shown in Figure 3. We observed the precursor protein F_0_ and the processed F_1_ subunit in the lysates of cells infected with the rCedV-NiV-B chimeras (Figure 3A) or the rCedV-HeV chimeras (Figure 3B,C). A distinct band representing the G glycoprotein was detected in all rCedV-NiV-B (Figure 3A) and rCedV-HeV (Figure 3B,C) infected lysates. Furthermore, the NiV-B and HeV F and G glycoprotein SDS-PAGE gel migration profiles were similar to those observed in the pcDNA3.1-NiV F + G (Figure 3A) and pcDNA3.1-HeV F + G (Figure 3B,C) transfected cell lysates, respectively. Whereas, HeV/NiV-B F_0_, F_1,_ or G bands were not observed in any of the rCedV infected lysates (Figure 3). The lower levels of F_0_, F_1,_ and G observed in the rCedV-HeV-Luc infected lysates in comparison to the other rCedV-HeV chimera infected lysates at 24 h post-infection (Figure 3B) could be attributed to slower virus replication kinetics of rCedV-HeV-Luc (see Figure 4B). To address this, we analyzed lysates of all rCedV-HeV chimeras at 48 h post-infection by western blot. As shown in Figure 3C, the levels of HeV F_0_, F_1,_ and G glycoproteins in all rCedV-HeV chimeras were comparable at this later time point. The presence of CedV N protein served as an expressed viral protein control and was observed in all infected cell lysates, while β-actin served as a lysate loading control. These data confirm the expression of NiV-B and HeV F and G glycoproteins in infected cells and indicate functional compatibility between rCedV and NiV-B and HeV envelope glycoproteins in a relevant viral context.

The replication kinetics of rCedV-NiV-B, rCedV-NiV-B-GFP, and rCedV-NiV-B-Luc (Figure 4A) and rCedV-HeV, rCedV-HeV-GFP, and rCedV-HeV-Luc (Figure 4B) were also compared to rCedV. Plaque assays were performed on harvested viral supernatants, and infectious virus titers were determined. We observed a gradual increase in infectious virus titers of all rCedV chimeric viruses that peaked 48 h post-infection (Figure 4). Specifically, the rCedV-NiV-B chimeras reached maximum virus titers of ~4–7 × 10^5^ PFU/mL, while the rCedV-HeV chimeras peaked at ~2–9 × 10^5^ PFU/mL. No statistically significant differences in replicated virus titers were observed between any of the rCedV-NiV-B chimeras or the rCedV-HeV chimeras or when compared to rCedV. In parallel, luciferase activity in rCedV-NiV-B-Luc and rCedV-HeV-Luc infected cells was measured. Figure 4 (right *y*-axes, black dashed lines) shows an increase in luminescence signal for both rCedV-NiV-B-Luc (Figure 4A) and rCedV-HeV-Luc (Figure 4B), which corresponded to the increase in infectious virus titers of their respective chimeras. Maximum luminescence signal was measured at 3.5 × 10^7^ RLU at 48 h post-infection for rCedV-HeV-Luc and at ~8 × 10^6^ RLU at 24 h post-infection for rCedV-NiV-B-Luc. The latter is likely due to extensive syncytia, and CPE observed in rCedV-NiV-B infected cells. These data illustrate that the rCedV chimeric viruses replicated efficiently and were comparable to rCedV and that luciferase activity is an indicator of viral genome expression.

### 3.3. Ephrin Entry Receptor Tropism of Recombinant Cedar Virus Chimeras

**Figure 5:**
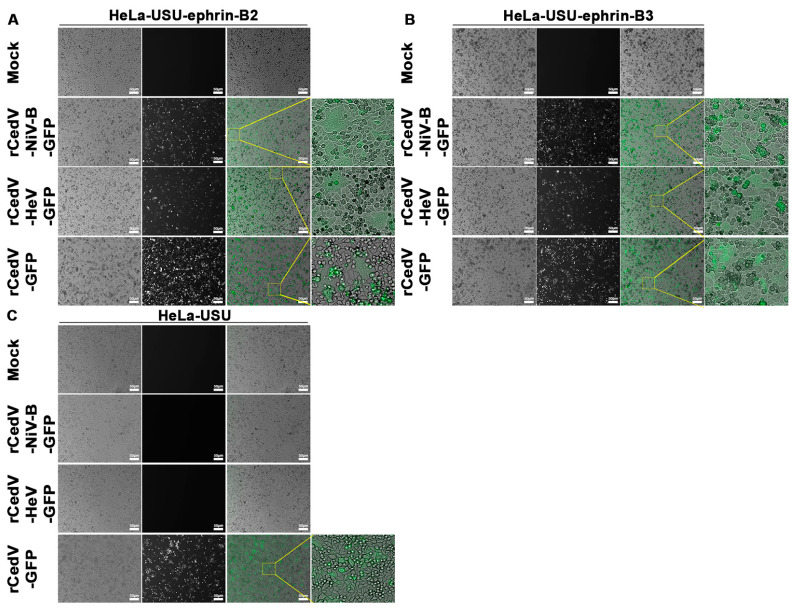
Ephrin-B2 and ephrin-B3 receptors facilitate rCedV-NiV-B-GFP and rCedV-HeV-GFP infection. Confluent HeLa-USU-ephrin-B2 (**A**), HeLa-USU-ephrin-B3 (**B**), and HeLa-USU (**C**) cells were uninfected (Mock) or infected with rCedV-NiV-B-GFP, rCedV-HeV-GFP, or rCedV-GFP at a MOI of 0.5. Infected cells were imaged for fluorescence and syncytia at 24 h post-infection. In each panel, transmitted light (1st column), fluorescence (2nd column), and merged (3rd column) images are shown. Zoomed-in regions are from the yellow boxes. Images were captured with a Zeiss Axio Observer A1 inverted microscope using a 5× objective. Representative images from two independent experiments are shown. Scale bar, 50 µm.

To define the receptor tropism of the newly generated rCedV chimeras, we used the NiV and HeV non-permissive cell line, HeLa-USU [31] and HeLa-USU cells stably expressing either ephrin-B2 (HeLa-USU-ephrin-B2) or ephrin-B3 (HeLa-USU-ephrin-B3) [20]. Here, all cells were either uninfected (Mock) or infected with rCedV-NiV-B-GFP, rCedV-HeV-GFP, or rCedV-GFP and at 24 h post-infection imaged for GFP expression. We observed fluorescence and/or syncytia in rCedV-NiV-B-GFP, and rCedV-HeV-GFP infected HeLa-USU-ephrin-B2 (Figure 5A) and HeLa-USU-ephrin-B3 (Figure 5B) cells, but not in the HeLa-USU infected cells (Figure 5C). In addition, GFP expression was detected in all rCedV-GFP infected cells, although syncytia were only observed in HeLa-USU-ephrin-B2 infected cells and were consistent with prior observations [20].

### 3.4. Recombinant Cedar Virus Chimeras Induce an Interferon Response

**Figure 6:**
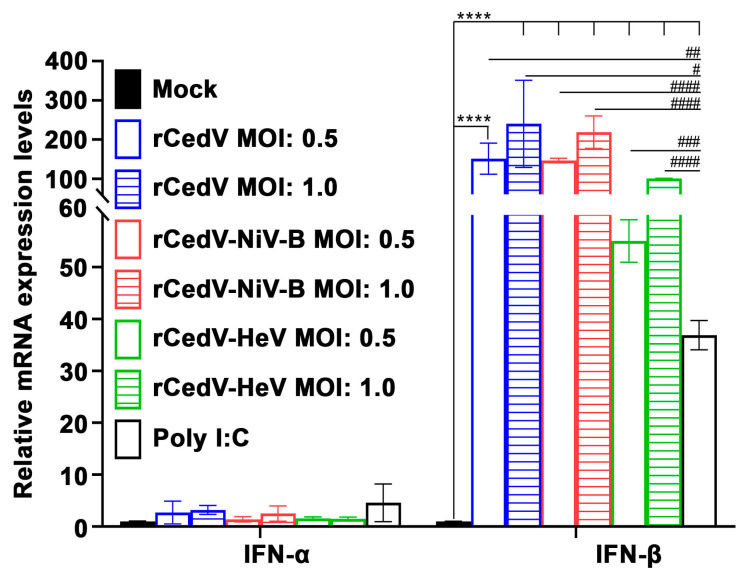
rCedV chimeras induce an IFN-β response. HeLa-CCL-2 cells were uninfected (Mock) or infected with rCedV-NiV-B, rCedV-HeV, or rCedV at a MOI of either 0.5 or 1.0 or transfected with Poly I:C (10 μg/mL) for 24 h. IFN-α and IFN-β mRNA expression were determined by qPCR. Fold changes were calculated relative to 18S ribosomal RNA and normalized to mock samples using the 2^(−ΔΔCt)^ method. These data represent mean ± standard deviation from two independent experiments, each performed in triplicate. Statistical analysis was performed with all samples in GraphPad Prism 9 by performing *t*-tests of each virus against Mock (asterisk *) or each virus against Poly I:C (hash #). **** *p* < 0.0001, # *p* = 0.011, ## *p* = 0.012, ### *p* = 0.0001 and #### *p* < 0.0001.

We next evaluated the induction of a type I IFN response in cells infected with the rCedV chimeras. HeLa-CCL-2 cells were uninfected (Mock) or infected with rCedV-NiV-B, rCedV-HeV, or rCedV. Additional HeLa-CCL-2 cells were transfected with Poly I:C to verify the induction of IFN-β (positive control). At 24 h post-infection, total RNA was extracted from all samples, and IFN-α and IFN-β mRNA levels were quantified by qPCR. As shown in Figure 6, in contrast to the mock samples, we observed a significant dose-dependent increase in IFN-β mRNA expression levels following rCedV-NiV-B, rCedV-HeV, and rCedV infection. A significant increase in the expression levels of IFN-α mRNA was not observed in any of the infected samples. These data demonstrate that rCedV chimeric viruses induced a robust and dose-dependent IFN-β response similar to rCedV [20] and also CedV [2].

### 3.5. Plaque Reduction Neutralization Test (PRNT) of Chimeric Recombinant Cedar Viruses by Cross-Reactive NiV and HeV Specific Monoclonal Antibodies

**Figure 7:**
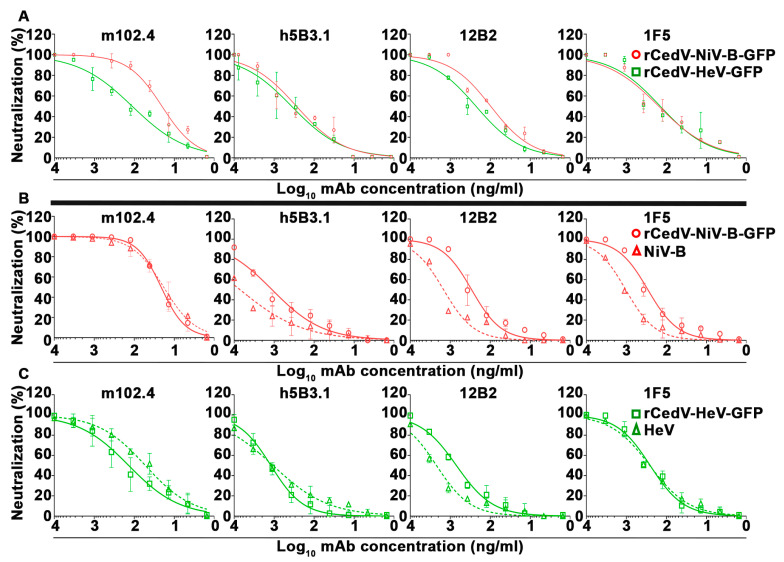
Neutralization of rCedV-NiV-B-GFP and rCedV-HeV-GFP by plaque reduction neutralization test (PRNT). Nine-point dose-response neutralization profiles for mAbs m102.4, h5B3.1, 12B2, and 1F5 against rCedV-NiV-B-GFP and rCedV-HeV-GFP at BSL-2 (**A**), at BSL-4 (**B**,**C**) and authentic NiV-B and HeV at BSL-4 (**B**,**C**). The diluted mAbs were incubated with an equal volume of either rCedV-NiV-B-GFP, rCedV-HeV-GFP, NiV-B, or HeV at an MOI of 0.0001 for 1 h at 37 °C, 5% CO_2_. MOI was calculated for a tentative 10^6^ cells/well and 0.4 mL virus and antibody mixture per well. Neutralization percent (%) was calculated based on PFU for each virus without mAb. These data represent mean ± standard deviation and are plotted as a non-linear regression curve fit with variable slope. BSL-2 studies are representative of two independent experiments, each performed in duplicate, and BSL-4 studies are from a single experiment performed in duplicate. The limit of detection for this assay was 50 PFU. Red circles and lines represent rCedV-NiV-B-GFP, red triangles and dashed lines represent NiV-B, green squares and lines represent rCedV-HeV-GFP, and green triangles and dashed lines represent HeV. The thick black line divides the BSL-2 PRNT from the BSL-4 PRNT.

To determine whether the rCedV-NiV-B-GFP and rCedV-HeV-GFP chimeras could serve as suitable surrogate viruses for authentic NiV-B and HeV, respectively, the ability of the GFP expressing rCedV chimeras to be neutralized by a panel of well-characterized NiV/HeV cross-reactive neutralizing mAbs was conducted by PRNT. The antibody panel included the human mAb m102.4 specific to the G glycoprotein and the humanized mAb h5B3.1 and murine mAbs 12B2 and 1F5 specific to the F glycoprotein [44,45,46,71]. Figure 7 illustrates the dose-response neutralization profiles for each mAb against rCedV-NiV-B-GFP and rCedV-HeV-GFP performed at BSL-2 (Figure 7A). In addition, a set of parallel PRNTs using authentic NiV-B and HeV and both rCedV chimeras were also performed simultaneously in BSL-4 containment (Figure 7B,C). Each mAb tested neutralized the infectivity of rCedV-NiV-B-GFP and rCedV-HeV-GFP and NiV-B and HeV (Figure 7) with highly similar dose-response virus neutralization profiles. The mean 50% inhibitory concentrations (IC_50_) for each of the mAbs against rCedV-NiV-B-GFP, rCedV-HeV-GFP, NiV-B, and HeV are summarized in Table 2. The most potent mAb was m102.4 with average IC_50_ values of ~20 ng/mL against rCedV-NiV-B-GFP and NiV-B, ~101 ng/mL against rCedV-HeV-GFP and ~50 ng/mL against HeV. The IC_50_ values for the mAbs tested here are within comparable ranges when compared to previous in vitro PRNT studies conducted with authentic NiV and HeV with the same set of cross-reactive neutralizing mAbs [43,44,46,66].

### 3.6. Correlation Analysis of Plaque Reduction Neutralization Tests (PRNT) Using GFP Expressing Recombinant Cedar Virus Chimeras and Authentic NiV-B and HeV

**Figure 8:**
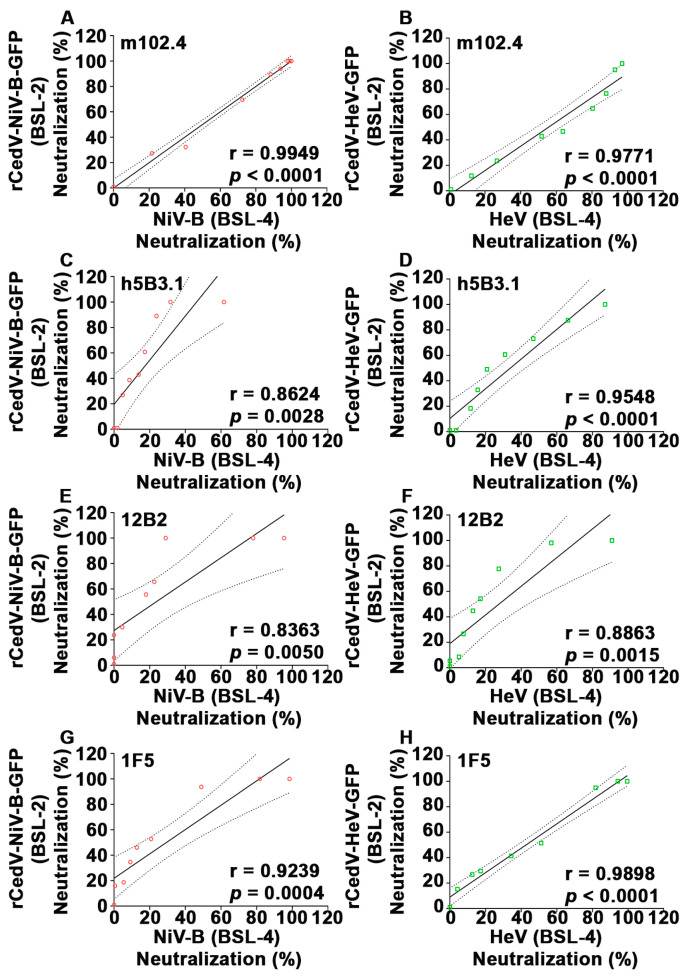
Correlation analysis of plaque reduction neutralization test (PRNT) neutralization values. Pearson correlation analysis of PRNT neutralization (%) values of rCedV-NiV-B-GFP versus NiV-B (**A**,**C**,**E**,**G**) and rCedV-HeV-GFP versus HeV (**B**,**D**,**F**,**H**) with mAbs m102.4, h5B3.1, 12B2 or 1F5. The Pearson correlation coefficient ‘r,’ *p*-value (two-tailed), linear regression line (solid lines), and 95% confidence intervals (dashed lines) are represented. Pearson’s r ≥ 0.8 and *p*-value < 0.05 indicate a strong significant positive correlation.

To further evaluate if the rCedV-NiV-B-GFP and rCedV-HeV-GFP chimeras are suitable surrogate virus platforms for authentic NiV-B and HeV antibody neutralization, a correlation analysis was performed. A Pearson correlation coefficient ‘r’ for each mAb was calculated by comparing the neutralization values derived from the rCedV chimeras BSL-2 PRNT with those of the NiV-B or HeV BSL-4 PRNT. The analysis indicated strong and statistically significant positive correlations between the two PRNTs (r values ranging from 0.86 to 0.99, *p* values from 0.005 to 0.0001) (Figure 8 and Table 3).

### 3.7. Establishment of a Fluorescence Reduction Neutralization Test (FRNT)

**Figure 9:**
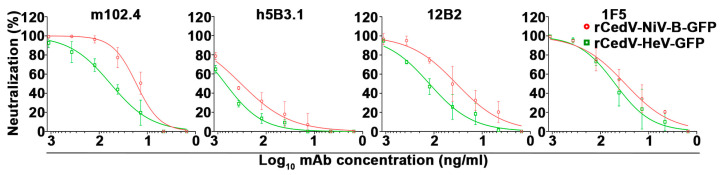
Neutralization profiles of NiV and HeV cross-reactive monoclonal antibodies by fluorescence reduction neutralization test (FRNT). Seven-point dose-response neutralization profiles for mAbs m102.4, h5B3.1, 12B2, and 1F5 against rCedV-NiV-B-GFP and rCedV-HeV-GFP. Neutralization percent (%) was calculated based on fluorescent foci for each virus without mAb. These data represent mean ± standard deviation from three independent experiments, each performed in triplicate. Data are plotted as non-linear regression curve fit with variable slope. The limit of detection for this assay was 50 fluorescent foci. Red circles and lines represent rCedV-NiV-B-GFP, and green squares and lines represent rCedV-HeV-GFP.

To further develop the utility of rCedV-NiV-B-GFP and rCedV-HeV-GFP chimeras as a surrogate platform for authentic NiV-B and HeV neutralization testing, we developed a high-throughput and quantitative assay based on the reduction in GFP fluorescent virus infection foci. Here, the virus neutralization efficacies of the same panel of mAbs used in the PRNT assays (Figure 7) were analyzed against rCedV-NiV-B-GFP and rCedV-HeV-GFP in a FRNT assay. As shown in Figure 9, the dose-response neutralization data were similar to those obtained by a PRNT (Figure 7A and Figure 9). The IC_50_ values for each mAb against rCedV-NiV-B-GFP and rCedV-HeV-GFP are summarized in Table 4. The mAb m102.4 potently neutralized rCedV-NiV-B-GFP at an IC_50_ of 16.91 ng/mL, while rCedV-HeV-GFP was neutralized by m102.4 and 1F5 with similar potencies at IC_50_ values of 58.12 ng/mL and 50.16 ng/mL, respectively. These data reveal that rCedV-NiV-B-GFP and rCedV-HeV-GFP mAb neutralization values in a FRNT are comparable to those obtained in a PRNT (comparisons of Table 2 and Table 4).

### 3.8. Correlation Analysis of the Conventional PRNT and the FRNT Neutralization Assays

**Figure 10:**
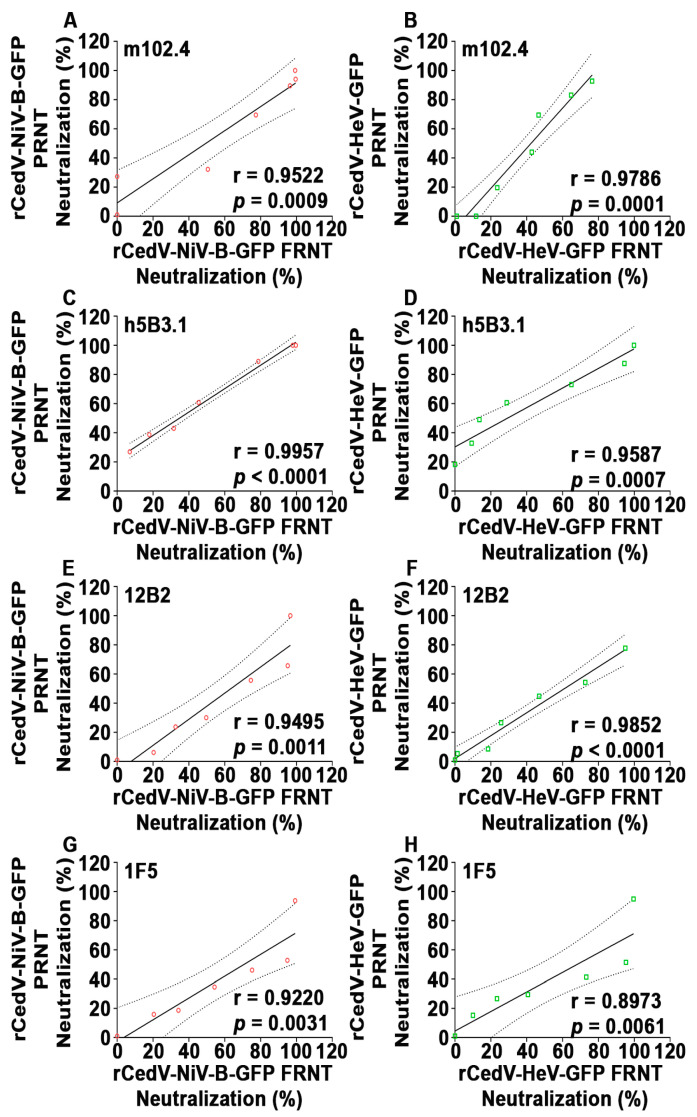
Correlation analysis of neutralization assays using the GFP expressing rCedV chimeric viruses. Pearson correlation analysis of neutralization (%) values from plaque reduction neutralization tests (PRNTs) (*y*-axes) and fluorescence reduction neutralization tests (FRNTs) (*x*-axes) performed with rCedV-NiV-B-GFP (**A**,**C**,**E**,**G**) and with rCedV-HeV-GFP (**B**,**D**,**F**,**H**) with mAbs m102.4, h5B3.1, 12B2 or 1F5. The Pearson correlation coefficient ‘r,’ *p*-value (two-tailed), linear regression line (solid lines), and 95% confidence intervals (dashed lines) are represented. Pearson’s r ≥ 0.8 and *p*-value < 0.05 indicate a strong significant positive correlation.

To further evaluate the FRNT assay as a suitable alternative virus neutralization assay to the standard PRNT, a correlation analysis was performed. A Pearson correlation analysis using the neutralization values obtained with each mAb against the rCedV-GFP chimeras by PRNT and FRNT assays was conducted (Figure 10 and Table 5), and a strong and significant positive correlation between the neutralization values obtained by PRNT versus the corresponding FRNT assay derived values was observed (r ≥ 0.9 and *p* ≤ 0.001). Taken together, these data demonstrate that rCedV-NiV-B-GFP and rCedV-HeV-GFP chimeric viruses are an ideal set of suitable surrogate viruses for authentic NiV-B and HeV for conducting a rapid FRNT-based assay for assessing NiV and HeV antibody neutralization.

### 3.9. Neutralization of rCedV-NiV-B-GFP and rCedV-HeV-GFP Using Henipavirus sG Immune Antisera

**Figure 11:**
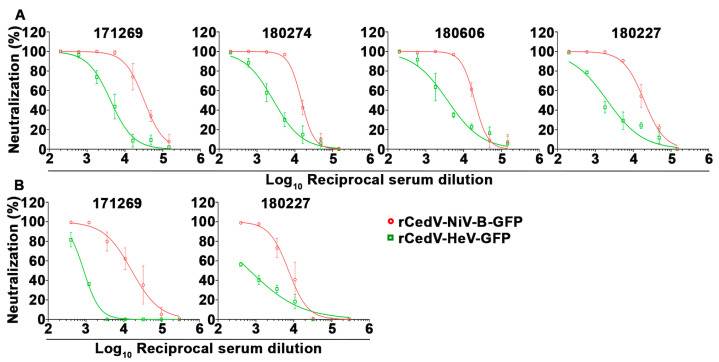
Neutralization profiles of NHP immunized sera against the GFP expressing rCedV chimeras by fluorescence reduction neutralization test (FRNT). Seven-point dose-response neutralization profiles of rhesus macaque sera collected on day 42 (**A**) and day 84 (**B**) post-immunization against rCedV-NiV-B-GFP and rCedV-HeV-GFP are shown. Neutralization percent (%) was calculated based on fluorescent foci for each virus without serum. These data represent mean ± standard deviation from two independent experiments, each performed in triplicate. Data are plotted as non-linear regression curve fit with variable slope. The limit of detection for this assay was 50 fluorescent foci. Animal ID numbers are 171269, 180274, 180606, and 180227. Red circles and lines represent rCedV-NiV-B-GFP, and green squares and lines represent rCedV-HeV-GFP.

The utility of the rCedV-NiV-B-GFP and rCedV-HeV-GFP viruses in the FRNT assay for measuring immune serum neutralization was examined by testing sera from NiV-sG or HeV-sG immunized nonhuman primates (NHP) (rhesus macaques) and rabbits, respectively. NHP subjects were immunized with an equal mixture of recombinant NiV-M and NiV-B sG glycoproteins (see Section 2) [47]. Figure 11 shows the dose-response neutralization profiles for each NHP sera against the rCedV-NiV-B-GFP and rCedV-HeV-GFP chimeras. The IC_50_ titers for each of the sera against rCedV-NiV-B-GFP and rCedV-HeV-GFP are summarized in Table 6. Sera collected on day 42 from subject 171269 had the highest neutralizing titer against rCedV-NiV-B-GFP (1:32,147) and rCedV-HeV-GFP (1:4157). In addition, we were also able to test sera collected on day 84 from 2 subjects, 171269 and 180227, and the IC_50_ titers are summarized in Table 6. Although still potently neutralizing, the 50% serum neutralization titers for animal 171269 declined 2-fold against rCedV-NiV-B-GFP to 1:16,101 and 4.8-fold against rCedV-HeV-GFP to 1:873.6. As expected, although the NHP sera were cross-neutralizing against rCedV-HeV-GFP the IC_50_ titers were much greater against the homologous immunized subjects (NiV).

**Figure 12:**
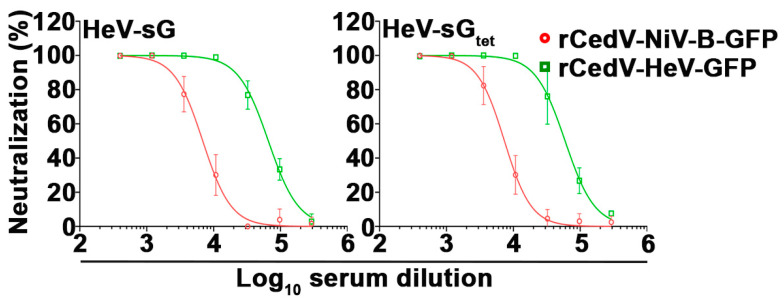
Neutralization profiles of rabbit immunized sera against rCedV chimeras expressing GFP. Seven-point dose-response neutralization profiles of HeV-sG (**left**) and HeV-sG_tet_ (**right**) immunized sera against rCedV-NiV-B-GFP and rCedV-HeV-GFP are shown. Neutralization percent (%) was calculated based on fluorescent foci for each virus without serum. These data represent mean ± standard deviation from two independent experiments, each performed in triplicate. Data are plotted as non-linear regression curve fit with variable slope. The limit of detection for this assay was 50 fluorescent foci. Red circles and lines represent rCedV-NiV-B-GFP, and green squares and lines represent rCedV-HeV-GFP.

We also analyzed sera from rabbits immunized with either HeV-sG or HeV-sG tetramer (HeV-sG_tet_) (see Materials and Methods). The sG immune rabbit sera neutralized the infectivity of both rCedV-NiV-B-GFP and rCedV-HeV-GFP, as shown by the dose-response neutralization profiles in Figure 12. The IC_50_ titers for each of the rabbit sera against rCedV-NiV-B-GFP and rCedV-HeV-GFP are summarized in Table 7. The HeV-sG and HeV-sG_tet_ sera had similar and very high neutralizing titers of 1:65,820 and 1:59,457, respectively, against rCedV-HeV-GFP. Here, the HeV-sG-specific rabbit sera had higher cross-neutralizing heterologous titers against rCedV-NiV-B-GFP (HeV-sG: 1:6881 and HeV-sG_tet_: 1:7367, respectively) with IC_50_ values again greater against the homologous immunized subjects (HeV), consistent with prior authentic NiV and HeV neutralization data derived from NiV-sG versus HeV-sG immunized cats [49].

## 4. Discussion

There has been increased concern regarding respiratory pathogens such as NiV as a consequence of the COVID-19 pandemic [72]. The most recent significant outbreak of NiV-B, which occurred in Kerala, India, in 2018, had a case-fatality rate of 91% and revealed a high incidence of acute respiratory distress syndrome among those infected, correlating with nosocomial respiratory droplet-mediated human-to-human transmission by exposure to patient’s coughing [73]. Experimentally, both NiV-M and NiV-B have been shown to cause lethal infection in NHPs when delivered as an aerosol [74,75], also the likely route of infection from deliberate release. Indeed, in 2020 the US Department of Health and Human Services (HHS) and Centers for Disease Control and Prevention (CDC) recommended that NiV be added to the list of Tier 1 Select Agents [76]. NiV-B has several characteristics enhancing its pandemic potential, including its respiratory tissue tropism; human susceptibility to infection; person-to-person transmission capability; and its potential to mutate, and the emergence of a human-adapted strain in South Asia could lead to the rapid spread of infection [77]. NiV and HeV have been important targets for vaccine development for more than 20 years, and these efforts have recently intensified [22,25].

In the present study, the rCedV reverse genetics platform [20,21] was modified by employing an optimized T7 promoter (T7_opt_) and the self-cleaving HHRbzA in the pOLTV5-rCedV antigenome plasmid, which improved the rescue efficiency of rCedVs. A similar strategy was used to rescue a number of other single-stranded, negative-sense RNA viruses, including NiV [36,78,79,80,81,82]. We then expanded the utility of the rCedV platform by replacing the coding sequences of the CedV F and G glycoproteins with their NiV-B or HeV counterparts to generate a panel of non-reporter gene and reporter gene encoding versions of rCedV-NiV-B and rCedV-HeV chimeric viruses. Interestingly, all chimeras appeared more fusogenic than those observed in rCedV-infected cells, and a similar phenotype was observed by Yeo et al., where cell fusion levels of CedV were consistently lower than NiV in transfected HEK293T cells [83]. All rCedV chimeras expressed the heterologous envelope glycoproteins in infected cells, replicated similarly in comparison to rCedV, and infection tropism was specific for ephrin-B2 and ephrin-B3 as entry receptors.

Previous in vitro cell-based assays demonstrated that CedV and rCedV induced a robust IFN-β response [2,20], and CedV infection stimulated the expression of interferon response genes, such as *IFNA7*, *CCL5, STAT1,* and *STAT2* in primary hamster endothelial cells [11]. Here, the rCedV chimeric viruses also induced the expression of IFN-β mRNA in an infection dose-dependent manner to comparable levels observed with rCedV infection and Poly I:C treatment.

Several surrogate NiV neutralization assays using recombinant Vesicular Stomatitis Virus (VSV) as a replication-incompetent pseudovirus with a deletion of the VSV G glycoprotein gene have been developed as a tool to measure NiV neutralization at BSL-2 containment [84,85,86]. The VSV-based pseudotype virus particle system has also been utilized with the HeV and GhV envelope glycoproteins for measuring neutralization [87]. Preparation of VSV pseudoviruses involves the budding progeny virions from cells that are transiently expressing henipavirus F and G glycoproteins which can sometimes be technically challenging to produce large quantities of pseudovirus stocks with reproducible characteristics. We previously found similar challenges in developing a retrovirus-based pseudotyped virus assay system that also required significant optimization [88]. These replication-incompetent pseudovirus assays are sensitive and have a high correlation when samples are scored as either positive or negative for henipavirus neutralizing antibody. However, specific mAb neutralization potencies or the virus-neutralizing titers of sera against NiV using VSV pseudotypes, as examples, are often quite different in comparison to sera titers obtained using authentic NiV [84,85,86].

Here, we sought to develop an improved surrogate neutralization assay system for NiV and HeV using rCedV as replication-competent chimeric viruses. The rCedV chimeric viruses developed and characterized here can be readily produced and stored in large quantities and are an authentic replication-competent henipavirus platform that can be used to study NiV and HeV F and G glycoprotein-mediated infection and also as surrogate viruses for authentic NiV and HeV in neutralization assays without the requirement for BSL-4 containment. Indeed, similar dose-response neutralization data and comparable IC_50_ concentrations of well-characterized NiV and HeV cross-reactive mAbs were observed between PRNTs using rCedV-NiV-B-GFP and NiV-B or rCedV-HeV-GFP and HeV. A strong and significant correlation between the overall neutralization values of the BSL-2 and BSL-4 PRNTs (Figure 8 and Table 3) validated the utility of the rCedV chimeric platform as suitable surrogate viruses for authentic NiV and HeV by PRNT.

We also expanded the utility of these novel reporter genes encoding rCedV chimeric viruses by developing a rapid and high-throughput fluorescence-based neutralization assay, FRNT. In contrast to the PRNT, which is the current gold standard for determining the presence of neutralizing antibodies and measuring the neutralizing titer in henipavirus-specific antisera, the FRNT (i) is high-throughput and allows for more samples to be assayed with more replicates in a 96-well plate format, (ii) requires smaller sample volumes, (iii) is less time consuming taking less than 36 h from infection to assay completion, and (iv) reduces the requirement for other reagents such as luciferase substrate. The rCedV-NiV-B-GFP and rCedV-HeV-GFP chimeric viruses were used to assess the neutralization potencies of mAbs by FRNT assay, and the neutralization values at each mAb concentration obtained by FRNT were found to be highly correlated with those values obtained by PRNT (Table 5). We further evaluated the utility of the rCedV-NiV-B-GFP and rCedV-HeV-GFP FRNT by measuring the neutralization activities of henipavirus sG immunized NHP sera and rabbit sera. The NiV-sG immunized NHP sera, and the HeV-sG immunized rabbit sera were both cross-neutralizing to rCedV-NiV-B-GFP and rCedV-HeV-GFP, with higher homotypic serum neutralization titers as expected. The rabbit HeV-sG immune sera exhibited greater heterologous neutralization titers in comparison to the NHP NiV-sG immune sera, which was also consistent with neutralization data derived from NiV-sG versus HeV-sG immunized cats against authentic NiV-M and HeV [49].

In summary, a surrogate henipavirus-based system for NiV and HeV using the rCedV platform suitable for use at BSL-2 containment has been developed and well-characterized. These rCedV chimeras can serve as useful tools to study NiV and HeV entry, membrane fusion mechanisms, and F and G glycoprotein interactions and aid in the discovery and development of henipavirus countermeasures. More importantly, the specificity and utility of the rCedV-NiV-B-GFP and rCedV-HeV-GFP viruses as a surrogate neutralization assay for authentic NiV and HeV to evaluate the neutralization potential of mAbs and NiV/HeV specific antisera has also been demonstrated. The rCedV chimeras will reduce the cost and technical challenges of the high-containment environment, particularly when large numbers of serum samples derived from NiV or HeV vaccine development programs will require testing and quantitation.

## 5. Patents

C.C.B. and M.A. are United States federal employees and co-inventors on US and foreign patents pertaining to Recombinant Cedar Virus Chimeras, whose assignees are the United States as represented by the Henry M Jackson Foundation for the Advancement of Military Medicine, Inc. (Bethesda, MD, USA).

## Figures and Tables

**Table 1:** GenBank accession numbers of NiV-B and HeV envelope glycoproteins.

Henipavirus	Isolate	Protein	GenBank Accession Number
NiV-B	2010 Faridpur	F	AEZ01396.1
		G	AEZ01397.1
HeV	2008 Redlands	F	AEQ38070.1
		G	AEQ38071.1

**Table 2:** IC_50_ values of NiV and HeV cross-reactive monoclonal antibodies against henipavirus infection by PRNT.

Monoclonal Antibody (mAb)	IC_50_ (95% CI) (ng/mL)					
	BSL-2	BSL-4		BSL-2	BSL-4	
	rCedV-NiV-B-GFP	rCedV-NiV-B-GFP	NiV-B	rCedV-HeV-GFP	rCedV-HeV-GFP	HeV
m102.4	20.30 (16.58–24.99)	21.20 (18.80–23.89)	18.36 (15.21–22.17)	112.9 (82.82–154.1)	137.0 (89.09–208.8)	52.41 (39.32–70.07)
h5B3.1	274.8 (185.9–403.7)	1122 (813.6–1548)	7101 (4323–15,087)	363.5 (241.6–546.3)	1202 (975.2–1481)	1064 (827.4–1372)
12B2	130.0 (97.10–174.0)	291.9 (219.9–381.5)	1467 (1098–1925)	502.3 (377.4–658.3)	700.1 (570.0–857.0)	2202 (1692–2846)
1F5	153.8 (107.0–219.4)	289.4 (229.9–360.9)	1036 (812.3–1298)	140.6 (83.29–232.0)	253.2 (200.5–318.8)	259.8 (213.2–315.8)

Note: All IC_50_ values are calculated by a nonlinear fit model and are shown with 95% confidence intervals (95% CI). BSL-2 studies are representative of two independent experiments, each performed in duplicate, and BSL-4 studies are from a single experiment performed in duplicate.

**Table 3:** Correlation analysis of rCedV chimeric viruses in BSL-2 PRNT versus NiV-B and HeV BSL-4 PRNT assays.

Virus	Monoclonal Antibody (mAb)	Pearson’s Correlation Coefficient (r)	Coefficient of Determination (R^2^)	Significance (*p*)	95% Confidence Interval (CI)
rCedV-NiV-B-GFP vs. NiV-B	m102.4	0.9949	0.9898	<0.0001	0.9750–0.990
	h5B3.1	0.8624	0.7437	0.0028	0.4640–0.9706
	12B2	0.8363	0.6994	0.0050	0.3873–0.9647
	1F5	0.9239	0.8536	0.0004	0.6722–0.9842
rCedV-HeV-GFP vs. HeV	m102.4	0.9771	0.9547	<0.0001	0.8914–0.9953
	h5B3.1	0.9548	0.9117	<0.0001	0.7945–0.9907
	12B2	0.8863	0.7855	0.0015	0.5400–0.9760
	1F5	0.9898	0.9796	<0.0001	0.9503–0.9979

Note: Correlation analysis was performed with the neutralization values from Figure 7.

**Table 4:** IC_50_ values of NiV and HeV cross-reactive specific monoclonal antibodies against rCedV-NiV-B-GFP and rCedV-HeV-GFP infection by FRNT.

Monoclonal Antibody (mAb)	IC_50_ (95% CI) (ng/mL)	
	rCedV-NiV-B-GFP	rCedV-HeV-GFP
m102.4	16.91 (14.72–19.45)	58.12 (49.27–68.70)
h5B3.1	333.0 (255.5–439.9)	700.2 (620.0–798.8)
12B2	34.07 (24.88–46.48)	124.5 (98.17–157.2)
1F5	28.97 (22.86–36.65)	50.16 (40.95–61.07)

Note: All IC_50_ values are calculated by a nonlinear fit model from three independent experiments, each performed in triplicate, and are shown with 95% confidence intervals (95% CI).

**Table 5:** Correlation analysis of rCedV chimeric viruses in the PRNT versus FRNT assays.

Virus	Monoclonal Antibody (mAb)	Pearson’s Correlation Coefficient (r)	Coefficient of Determination (R^2^)	Significance (*p*)	95% Confidence Interval
rCedV-NiV-B-GFP	m102.4	0.9522	0.9067	0.0009	0.7038–0.9931
	h5B3.1	0.9957	0.9914	<0.0001	0.9698–0.9994
	12B2	0.9495	0.9016	0.0011	0.6894–0.9927
	1F5	0.9220	0.8501	0.0031	0.5527–0.9886
rCedV-HeV-GFP	m102.4	0.9786	0.9576	0.0001	0.8573–0.9970
	h5B3.1	0.9587	0.9191	0.0007	0.7397–0.9941
	12B2	0.9852	0.9707	<0.0001	0.8997–0.9979
	1F5	0.8973	0.8051	0.0061	0.4448–0.9849

Note: Correlation analysis was performed with the neutralization values from Figure 7A and Figure 9.

**Table 6:** IC_50_ Anti-NiV G glycoprotein immunized rhesus macaque serum titers against rCedV-NiV-B-GFP and rCedV-HeV-GFP infection.

Animal ID	IC_50_ (95% CI) (Serum Titer)	
	rCedV-NiV-B-GFP	rCedV-HeV-GFP
171269 Day 42	1:32,147 (1:29,414–1:35,182)	1:4157 (1:3711–1:4658)
171269 Day 84	1:16,101 (1:12,288–1:21,024)	1:873.6 (1:809.4–1:941.3)
180274 Day 42	1:14,860 (1:14,018–1:15,761)	1:2704 (1:2375–1:3082)
180606 Day 42	1:19,480 (1:18,181–1:20,948)	1:3739 (1:3094–1:4542)
180227 Day 42	1:19,408 (1:17,817–1:21,158)	1:2048 (1:1668–1:2539)
180227 Day 84	1:7283 (1:6079–1:8739)	1:689.0 (1:499.2–1:904.4)

Note: All IC_50_ values are calculated by a nonlinear fit model from two independent experiments, each performed in triplicate, and are shown with 95% confidence intervals (95% CI).

**Table 7:** IC_50_ rabbit serum titers against rCedV-NiV-B-GFP and rCedV-HeV-GFP infection.

Immunogen	IC_50_ (95% CI) (Serum Titer)	
	rCedV-NiV-B-GFP	rCedV-HeV-GFP
HeV-sG	1:6881 (1:6211–1:7616)	1:65,820 (1:61,407–1:70,409)
HeV-sG_tet_	1:7367 (1:6642–1:8158)	1:59,457 (1:54,002–1:65,333)

Note: All IC_50_ values are calculated by a nonlinear fit model from two independent experiments, each performed in triplicate, and are shown with 95% confidence intervals (95% CI).

## Data Availability

Not applicable.

## Supplementary Materials

The following supporting information can be downloaded at: https://www.mdpi.com/article/10.3390/v15051077/s1, Table S1: Mutations identified by genome sequencing of recombinant Cedar virus chimeras.
