# Supplementary material for: A Recombinant Chimeric Cedar Virus-Based Surrogate Neutralization Assay Platform for Pathogenic Henipaviruses
Source: Viruses. 2023 Apr 28;15(5):1077. doi: 10.3390/v15051077 (PMC10223282; doi:10.3390/v15051077)
Supplement: Supplementary file 1 [file viruses-15-01077-s001.zip › viruses-2231740-supplementary.pdf]

Supplement: Amaya, M., et al., A Recombinant Chimeric Cedar virus Based Surrogate Neutralization Platform for Pathogenic Henipaviruses. *Viruses* 2023.

Table S1. Mutations identified by genome sequencing of recombinant Cedar virus chimeras.

**Virus**	**Total # of changes**	**Non coding mutations**				**Coding mutations**			
		Location		base pair (bp) change	amino acid (aa) change	Location (aa)	Protein	base pair (bp) change	amino acid (aa) change
rCedV-NiV-B	4					445	N	AGC-GGC	Ser-Gly
						296	M	AGG-GGG	Arg-Gly
						885	L	CAA-CGA	Gln-Arg
						2379	L	TTT-TCT	Phe-Ser
rCedV-NiV-B-GFP	8	bp 7	3' Le	C-A	N/A	442	N	ATA-ACA	Ile-Thr
		aa 507	N protein	GAT-GAC	Asp-Asp	505	N	TTC-CTC	Phe-Leu
		bp 1866	N stop IGR	T-C	N/A				
		bp 1867	N stop IGR	T-C	N/A				
		bp 1915	N stop IGR	T-C	N/A				
		aa 1528	L protein	TCA-TCG	Ser-Ser				
rCedV-NiV-B-Luc	9	bp 7	3' Le	C-A	N/A	441	N	TCA-CCA	Ser-Pro
		bp 1737	N stop IGR	T-C	N/A	231	Luc	CAG-CCG	Gln-Pro
		bp 1742	N stop IGR	T-C	N/A	25	M	GAA-AAA	Glu-Lys
		bp 1783	N stop IGR	T-C	N/A	2109	L	AAA-GAA	Lys-Glu
		bp 1894	N stop IGR	T-C	N/A				
rCedV-HeV	2	bp 1755	N stop IGR	C-A	N/A	546	F	ACA-ATA	Thr-Ile
rCedV-HeV-GFP	3	bp 20	3' Le	G-A	N/A	445	N	AGC-GGC	Ser-Gly
						245	M	ATG-CTG	Met-Leu
rCedV-HeV-Luc	5	bp 2	3' Le	C-T	N/A				
		bp 3	3' Le	C-T	N/A				
		bp 4	3' Le	A-T	N/A				
		bp 7	3' Le	C-A	N/A				
		bp 19767	5' Tr	A-T	N/A				

Abbreviations: rCedV-NiV-B, recombinant Cedar virus with the fusion and attachment glycoproteins replaced with those of Nipah virus Bangladesh; rCedV-HeV, recombinant Cedar virus with the fusion and attachment glycoproteins of Hendra virus; bp, base pair; aa, amino acid; 3′ Le, 3′ leader; N, nucleoprotein; GFP, green fluorescent protein; Luc, luciferase protein; M, matrix protein; F, fusion glycoprotein; L, polymerase protein; IGR, intergenic region; 5′ Tr, 5′ trailer; A, adenine; T, thymine; C, cytosine; G, guanine; Ser, serine; Gly, glycine; Arg, arginine; Gln, glutamine; Phe, phenylalanine; Ile, isoleucine; Thr, threonine; Leu, leucine; Pro, proline; Glu, glutamic acid; Lys, lysine; Met, methionine; Asp, aspartic acid; #, number; N/A, not applicable.
